# Collective pharmaceutical procurement in China may have unintended consequences in supply and pricing

**DOI:** 10.7189/jogh.10.010314

**Published:** 2020-06

**Authors:** Shan Jiang, Zhuo Chen, Tao Wu, Hui Wang

**Affiliations:** 1 School of Population and Public Health, University of British Columbia, Vancouver, BC, Canada; 2 Department of Health Policy and Management, College of Public Health, University of Georgia, Athens, Georgia, USA; 3 School of Economics, Faculty of Humanities and Social Sciences, University of Nottingham Ningbo China, Ningbo, China; 4 Shanghai Jiao Tong University School of Medicine, Shanghai, China; 5 School of Public Health, Shanghai Jiao Tong University School of Medicine, Shanghai, China

The collective pharmaceutical procurement was launched in China in 2018 to reduce the prices of selected drugs, by pooling the demands of member cities and granting the contract to the manufacturer with the lowest bid. We found the procurement significantly decreased the prices of most drugs. We also identified significant price increases on some drugs, indicating that manufacturers of these drugs may have strong market power to manipulate prices. The “winner-takes-all” principle applied in the procurement may further increase the market power of winning manufacturers by expanding their respective market shares. They may take the advantage of the market power to increase drug prices in the long-run. The continuously lowering price-caps may force the losing bidders to exit the market. A careful assessment of the unintended consequences of the collective procurement is warranted.

In many provinces of China, hospitals and pharmacies have been conducting the group procurement of medicines since 2010s, to increase the bargaining power on price negotiation, as recommended by the then Ministry of Health, China. Provincial health authorities organized the group procurement and adopted price-caps to regulate the negotiations. While the public criticized the price-caps for being too high, the National Healthcare Security Administration launched in December 2018 a nationwide collective pharmaceutical procurement pilot trial with lower price-caps, coded as “4 + 7”, where the demands for a certain drug of member cities across China were pooled and granted to the manufacturer with the lowest bid, if it was below the price-cap [1]. The code “4 + 7” comes from the initial group of cities that include four provincial-level metropolitans (Beijing, Shanghai, Tianjin, and Chongqing) and seven major cities (Shenyang, Dalian, Xiamen, Guangzhou, Shenzhen, Chengdu, and Xi’an).

To investigate the impact of the “4 + 7” procurement, we compared the list of medicines in the procurement plan and the final contracted list to assess the completion of the procurement. We then retrieved the prices of the contracted drugs in Shanghai before and after the procurement, to compare the price change [2]. We also collected the sales volumes of the manufacturers who gained the “4 + 7” contracts before 2018 as additional background information [3]. Shanghai was chosen for this analysis because of better data transparency and availability compared with other member cities. Since the previous procurement of drugs were contracted at different prices in different batches before the “4 + 7” pilot, we weighted the prices of each drug per batch by the purchased amounts to construct a weighted average composite price. The composite price was then compared with the agreement price in the “4 + 7” procurement (**Table 1**).

**Table 1:** Drug price change due to the “4 + 7” collective pharmaceutical procurement and associated providers.

No.	Drug	Format	Price before 4 + 7 (CNY, USD) *	4 + 7 contract price, (CNY, USD)†	Price change	Contractor‡	Market share§, %	Market share rank‖
1	Atorvastatin	Tablet	¥5.00, $0.76	¥0.94, $0.14	-81.1%	Jialin	17.42	2
2	Rosuvastatin	Tablet	¥5.98, $0.90	¥0.78, $0.12	-87.0%	Jingxin	6.91	3
3	Clopidogrel	Tablet	¥3.68, $0.56	¥3.18, $0.48	-13.6%	Salubris	30.13	2
4	Irbesartan	Tablet	¥3.04, $0.46	¥0.20, $0.03	-93.4%	Huahai	0.61	11
5	Amlodipine	Tablet	¥1.88, $0.28	¥0.15, $0.02	-92.1%	Jingxin	0.02	23
6	Entecavir	Tablet	¥28.06, $4.24	¥0.62, $0.09	-97.8%	Chia Tai-Tianqing	44.22	1
7	Escitalopram	Tablet	¥13.94, $2.11	¥4.42, $0.67	-68.3%	KELUN	9.93	3
8	Paroxetine	Tablet	¥4.26, $0.64	¥1.67, $0.25	-60.8%	Huahai	41.47	2
9	Olanzapine	Tablet	¥24.03, $3.63	¥9.64, $1.46	-59.9%	Hansoh	59.99	1
10	Cefuroxime	Tablet	¥0.60, $0.09	¥0.51, $0.08	-14.2%	Beite	2.63	7
11	Risperidone	Tablet	¥3.24, $0.49	¥0.17, $0.03	-94.8%	Huahai	5.73	6
12	Gefitinib	Tablet	¥63.00, $9.52	¥54.70, $8.26	-13.2%	Qilu Hainan	11.45	2
13	Fosinopril	Tablet	¥2.80, $0.42	¥0.84, $0.13	-69.9%	Squibb	82.81	1
14	Irbesartan/Hydrochlorothiazide	Tablet	¥4.40, $0.66	¥1.09, $0.16	-75.2%	Huahai	4.72	4
15	Lisinopril	Tablet	¥0.26, $0.04	¥0.23, $0.03	-11.6%	Huahai	35.68	1
16	Tenofovir	Tablet	¥6.67, $1.01	¥0.59, $0.09	-91.1%	Beite	4.02	3
17	Losartan	Tablet	¥5.60, $0.85	¥1.05, $0.16	-81.3%	Huahai	6.57	3
18	Enalapril	Tablet	¥0.22, $0.03	¥0.56, $0.08	150.4%	Yangzijiang	76.58	1
19	Levetiracetam	Tablet	¥3.78, $0.57	¥2.40, $0.36	-36.5%	Jingxin	0.43	3
20	Imatinib	Tablet	¥45.00, $6.80	¥10.40, $1.57	-76.9%	Hansoh	12.35	2
21	Montelukast	Tablet	¥6.40, $0.97	¥3.88, $0.59	-39.4%	Minsheng	2.71	4
22	Montmorillonite	Granule	¥1.84, $0.28	¥0.68, $0.10	-63.1%	Simcere	3.71	3
23	Pemetrexed	Injection	¥1160.20, $175.26	¥2776.97, $419.48	139.4%	Huiyu	4.28	4
24	Flurbiprofen	Injection	¥12.82, $1.94	¥21.95, $3.32	71.3%	Tide	98.33	1

*CNY, Chinese Yuan (¥). USD, US Dollar ($). Composite price per unit before “4 + 7” procurement, converted to 2018 CNY and USD according to currency exchange rate and consumer price index (CPI).

†Composite price per unit in “4 + 7” contract, converted to 2018 CNY and USD according to currency exchange rate and CPI.

‡Beite, Chengdu Beite Pharmaceutical Industry. Chia Tai-Tianqing, Chia Tai-Tianqing Pharmaceutical Holdings Co., Ltd Hansoh, Jiangsu Hansoh pharmaceuticals. Huahai, Zhejiang Huahai Pharmaceutical Co., Ltd Huiyu, Sichuan Huiyu Pharmaceutical Limited. Jialin, Beijing Jialin Pharmaceutical Industry Company Limited. Jingxin, Zhejiang Jingxin Pharmaceutical Co. Ltd KELUN, Sichuan KELUN PHARMACEUTICAL Co., Ltd Minsheng, Hangzhou Minsheng Binjiang Pharmaceuticals. Qilu Hainan, Qilu Pharmaceutical Hainan Co., Ltd Salubris, Shenzhen Salubris Pharmaceuticals. Simcere, Hainan Simcere Pharmaceuticals. Squibb, Sino-American Shanghai Squibb Pharmaceuticals Ltd Tide, Beijing Tide Pharmaceutical Co., Ltd Yangzijiang, Yangzijiang Pharmaceuticals.

§Market share, sales volume proportion of contractor in the separate drug market.

‖Market share rank, the rank of sales volume of contractor in the separate drug market.

Negotiation for 25 out of 31 drugs successfully resulted in a contract price, indicating a completion rate of 81%. The demand and supply sides failed to achieve an agreement on the remaining 6 drugs (ie, Amoxicillin, Azithromycin tablet, Tramadol, Alfacalcidol, Captopril, Azithromycin injection). We were able to retrieve the prices of 24 medications among the contracted ones except Dexmedetomidine, and found that the prices of 21 medications decreased, with a range from −11.6% to −97.8%. On the contrary, the prices of three drugs (ie, Enalapril, Pemetrexed, and Flurbiprofen) increased by 150.4%, 139.4%, and 71.3%, respectively. The weighted average of price change for the 24 medications was −40.0%.

The collective medical procurement decreased the drug prices significantly on average. However, we found that, in six cases of the 24 contracted medications (ie, Entecavir, Olanzapine, Fosinopril, Lisinopril, Enalapril, Flurbiprofen), the contract was obtained by the manufacturer with biggest market share (ie, largest sales volume proportion) in a separate market before 2018 (**Table 1**). In some other cases, major manufacturers (eg, Huahai, Jingxin, Beite, Hansoh) obtained several contracts for multiple products. For example, Huahai gained contracts for Irbesartan, Paroxetine, Risperidone, Irbesartan/Hydrochlorothiazide, Lisinopril, and Losartan. Therefore, the impacts of the collective procurement need to be further evaluated carefully for three reasons. First, the collective procurement applies a “winner-takes-all” principle, which may increase the market power of the winning manufacturers in each drug market. A manufacturer with market power close to monopoly will in turn weaken the bargaining power of hospitals and pharmacies in the long run [4]. The monopoly power may impede government’s attempt to reach an agreed contract at a low price which may result in same or even higher drug prices. In contrast to the “winner-takes-all” principle, state Medicaid agencies in the US have managed the market by awarding contracts to more than one provider, carving up markets in regions to ensure all qualified parties shared in the contracts [5].

**Figure:**
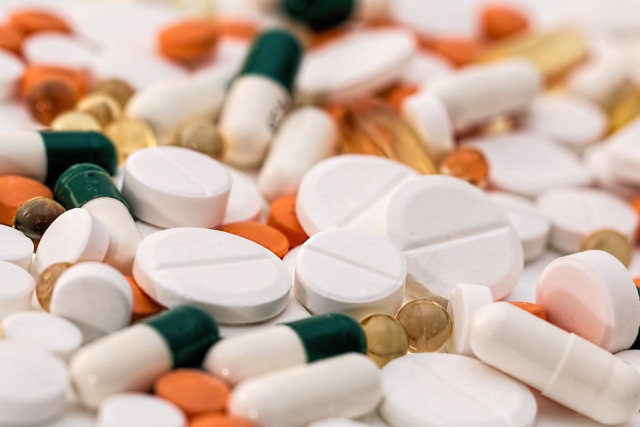
Photo: Downloaded from https://pixabay.com/zh/photos/headache-pain-pills-medication-1540220/.

Second, continuously lowering the price-cap levels may force some manufacturers to exit from the market, especially the firms producing generic drugs [6]. In Canada, generic manufacturers became more likely to exit market when the price-caps were set at 25% of the brand-name product prices in 2010 than when the price-caps at 70% of brand-name prices in late 1990s [6]. Although market entries and exits result from market competition regularly, forced exits are a concern because it may lead to a loss of consumer welfare in the long term. A good example is that US Food and Drug Administration (FDA) promulgated laws to attract more generic providers by expediting the review of generic drug applications and reducing the dominance of brand-name manufacturers that hinders market competition [7]. We notice that some manufacturers in previous provincial procurements did not participate in the “4 + 7”. Though we do not have access to the information that may explain the exits and do not know whether the exits are temporary, government need to be cautious of forced exits of generic manufacturers due to continuously lowered price-caps.

Third, manufacturer participants in the procurement may sacrifice some revenues to become the “winners” on certain drugs. Consequently, they may have the incentives to increase the prices of other medical products (eg, drugs or active pharmaceutical ingredients) that are not covered by the National Essential Medicines List or public funded health insurance to make up their loss in “4 + 7”. Strong price control of drug manufacturers will naturally lead to market-dominance behaviors [8]. The market-dominance behaviors may indirectly or directly increase the health expenditures by payers or patients.

Hence, while collective drug procurement is an important policy tool, its long-term impact may need to be carefully assessed and accounted for in policymaking.
